# Editorials

**Published:** 1911-06

**Authors:** 


### EDITORIALS

The Business Office of the *Journal-Record* is 1 1-2 to 5 1-2 South Broad Street.
The Editorial Office is 1014-15 Century Building.
Address all Business Communications to *Journal-Record of Medicine*, 1 1-2 to 5 1-2 South Broad Street.
Make remittances payable *to The Journal-Record of Medicine.*
On matters pertaining to the Editorial and Original Communications, address Edgar G. Ballenger, M. D., Atlanta, Ga.
Reprints of Original Articles will be furnished at cost price. Requests for the same should always be made in THE MANUSCRIPT.
We will present, postpaid, on request, to each contributor of an original article, twenty (20) marked copies of *The Journal-Record of Medicine* containing such article.

### CHEER UP.

Some time ago the writer published an article on optimistic therapeutics, which many members of the profession have been kind enough to commend.

This thought has continued to grow until the conviction has forced itself that it is not only to the physician's interest, but his absolute duty to cultivate an optimistic attitude towards his patients and their ailments.

Gloomy prognoses are sometimes forced on us, and at times we cannot logically understand how a recovery can take place. Still, occasionally, under just such circumstances and to our intense surprise, some hidden dynamic gets busy, bringing about marvelous changes—changes we cannot explain, but at which we can only wonder.

Such lessons teach us that no doctor should utter a dictum as of a supreme court, lest the *vis naturae medicatrix* reverse him to his discomfiture.

The wise statesmen and jurists of our land, realizing the fallibility of human discrimination, have arranged for a series of appeals from one tribunal to another, so that the chances of injustice to any individual is reduced to a minimum.

On this principle we should, in serious cases, always encourage an appeal, if practicable, and only under the most exceptional and desperate circumstanes should any medical attendant assume a pessimistic state of mind which would tend to close the door of hope; and, while reserving a loop-hole for escape from possible errors of judgement, an habitual optimistic attitude will more often prove a winner than a loser.

---

### PELLAGRA

The paper on Pellagra appearing in this month's Journal, deserves consideration. While the Journal does not take either side of the question, it should be read with considerable interest. The paper shows that there has been considerable time and thought spent and while Pellagra, today stands without etymology and while new theories are advancing each year, we are still in doubt as to its real cause.

No doubt food stuffs play an important roll. Just what foods or what sort of foods go to bring about these causes, we are absolutely in darkness, but evidently there has been something brought to play, especially in the South in the last fifteen years, which is causing so much disaster and if in the next quarter of a century Pellagra still holds its own as at the present time or advance with increased gravity, it will stand as the most frightful disease with which we have to deal.

Whatever the cause may be it should be studied with increased fortitude and the work should not cease until the real cause is determined. If cotton seed oil products are in any way responsible for the condition, it would be much easier to combat, than if some of the old theories are true.

